# Seven Years of Participation Churn in the Medicare Quality Payment Program

**DOI:** 10.1001/jamanetworkopen.2025.32838

**Published:** 2025-09-19

**Authors:** Meng-Yun Lin, Kathleen Carey, Risha Gidwani, Amresh D. Hanchate

**Affiliations:** 1 Department of Social Sciences and Health Policy, Wake Forest University School of Medicine, Winston-Salem, North Carolina; 2 Department of Health Law, Policy and Management, Boston University School of Public Health, Boston, Massachusetts; 3 Division of Health Care Policy and Research, University of Colorado School of Medicine, Aurora; 4 RAND Corporation, Santa Monica, California

## Abstract

This cohort study examines participation patterns in the Medicare Quality Payment Program to evaluate understudied obstacles to the Centers for Medicare & Medicaid Services goal of transitioning to value-based payment by 2030.

## Introduction

The Centers for Medicare & Medicaid Services (CMS) launched the Quality Payment Program (QPP) in 2017 to shift Medicare from fee-for-service to value-based payments.^1^ Clinicians can participate through 2 tracks: the Merit-Based Incentive Payment System (MIPS), which adjusts payments on the basis of reported performance without financial risk, or Advanced Alternative Payment Models (A-APM), which require downside risk but offer higher potential financial rewards.^2^ Each year, clinicians may switch tracks or change reporting modes within MIPS. We examined participation patterns to evaluate this churn—an understudied obstacle to CMS’s goal of transitioning all Medicare fee-for-service practitioners to value-based payment by 2030.^3^

## Methods

This cohort study used the Medicare Provider Utilization and Payment Data Physician and Other Supplier Public Use File^4^ to identify clinicians who billed Medicare Part B services between 2017 and 2023, including physicians, nurse practitioners, physician assistants, and other clinicians. Using National Provider Identifiers, we linked these data to the QPP Experience Report^5^ to determine MIPS participation status and reporting modes (individual clinicians, group practice or virtual group, or MIPS APM). Clinicians not participating in MIPS were either QPP-ineligible or enrolled in the A-APM track (see eMethods in Supplement 1 for eligibility and track descriptions).

Each clinician was classified annually as nonparticipant, individual, group, or MIPS APM. We tracked yearly transitions into and out of MIPS beginning in 2018. Additionally, we identified clinicians who never participated, participated continuously, or switched tracks. For switchers, we counted number of transitions and examined switch direction (exited or entered MIPS) among 1-time switchers. Finally, we assessed changes in reporting mode among continuous MIPS participants.

Clinicians were followed for up to 7 years. We used descriptive statistics to summarize longitudinal patterns of MIPS participation. Analyses were conducted using Stata statistical software version 18 (StataCorp). This study was deemed exempt from review and the need for informed consent because the data were deidentified and publicly available, in accordance with 45 CFR §46, by the Wake Forest University School of Medicine institutional review board. We followed the STROBE reporting guidelines.

## Results

We identified 1 587 280 unique Medicare clinicians from 2017 to 2023. During this period, MIPS participation declined substantially, from 671 928 of 1 026 824 (65.4%) to 427 266 of 1 196 535 (35.7%) (Figure). Among MIPS participants, the proportion reporting through APM entities decreased from 195 544 of 671 928 (29.1%) to 96 478 of 427 266 (22.6%), and individual participation declined from 102 973 of 671 928 (15.3%) to 42 109 of 427 266 (9.9%). In contrast, group-based reporting increased from 373 411 of 671 928 (55.6%) to 319 739 of 427 266 (67.5%). Between 2017 and 2018, 146 375 of 1 060 686 (13.8%) of clinicians exited MIPS, while only 56 216 of 1 060 686 (5.3%) joined.

**Figure.**
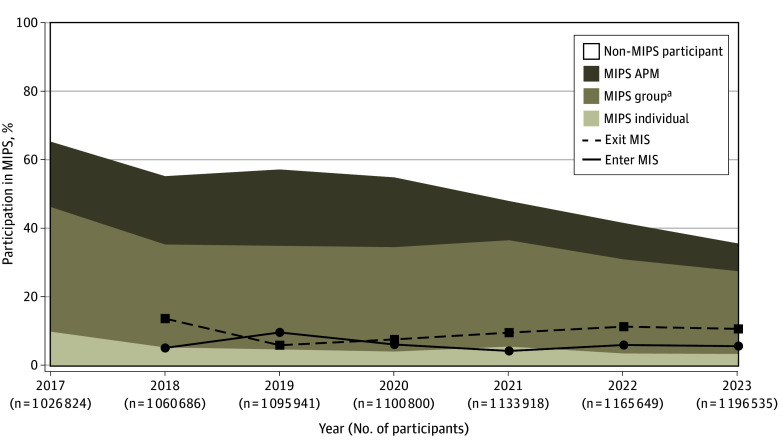
Trends in Merit-Based Incentive Payment System (MIPS) Participation and Transitions Under the Quality Payment Program, 2017 to 2023 The stacked area chart illustrates participation trends of Medicare practitioners in the MIPS track from 2017 to 2023. Practitioners are categorized by their MIPS participation and reporting mode: non-MIPS participant, MIPS individual, MIPS group, or MIPS Alternative Payment Model (APM). The solid line shows the percentage of practitioners entering the MIPS track, while the dashed line shows those exiting in subsequent years. ^a^MIPS group includes group participants, virtual group participants (n = 453 in 7 years), and subgroup participants (n = 92 in 2023).

Over the period, 357 693 clinicians (22.5%) participated in MIPS continuously, while 512 498 (32.3%) never participated (Table). The remaining 717 089 clinicians (45.2%) switched tracks at least once. Among these switchers, 489 549 (68.3%) transitioned only once—most commonly leaving MIPS permanently (351 524 clinicians [71.8%]) rather than joining (138 025 clinicians [28.2%]). A total of 164 160 clinicians (22.9%) switched twice, and 63 380 (8.8%) were frequent switchers (eg, ≥ 3 transitions). Among clinicians with continuous MIPS participation, 227 862 (63.7%) maintained the same reporting mode, while 76 180 (21.3%) changed once and 53 651 (15.0%) changed multiple times. Of those who never changed reporting mode, the majority (161 095 clinicians [70.7%]) participated through a group practice, followed by 53 627 (23.5%) via an APM entity and 13 140 (5.8%) as individual clinicians.

**Table.** Longitudinal Patterns in MIPS Engagement and Reporting Modes Among Clinicians Billing Medicare Part B Services, 2017 to 2023

Characteristic	Clinicians, No. (%) (N = 1 587 280)
Never participated in MIPS	512 498 (32.3)
Switched between MIPS and A-APM tracks	717 089 (45.2)
Continuous MIPS participants	357 693 (22.5)
Among practitioners who switched tracks (n = 717 089)	
1-Time switchers	489 549 (68.3)
2-Time switchers	164 160 (22.9)
Frequent switchers^a^	63 380 (8.8)
Among 1-time switchers (n = 489 549)	
Exit MIPS	351 524 (71.8)
Enter MIPS	138 025 (28.2)
Among continuous MIPS participants (n = 357 693)	
Never changed reporting mode	227 862 (63.7)
Changed mode once	76 180 (21.3)
Changed mode multiple times	53 651 (15.0)
Among continuous MIPS participants who never changed mode (n = 227 862)	
Individual	13 140 (5.8)
Group	161 095 (70.7)
MIPS APM	53 627 (23.5)

Abbreviations: A-APM, Advanced Alternative Payment Models; MIPS, Merit-Based Incentive Payment System.

^a^ Frequent switcher means 3 or more switches between MIPS and A-APM.

## Discussion

Using most recent QPP data, this cohort study found that a substantial proportion of clinicians departed MIPS each year, consistently outpacing entrants. Exiters included clinicians who transitioned to A-APM, became ineligible for QPP due to low volume, or retired. A recent MedPAC report documented a 4-fold increase in A-APM participation from 2017 to 2022,^6^ suggesting that many exits reflected movement toward A-APM, aligning with CMS goals.

Despite signs of progress, concerns remain. Over one-fifth of clinicians remained in MIPS, and nearly one-half switched tracks 1 or more times. Similarly, more than one-third of continuous MIPS participants changed reporting mode. Persistent MIPS participation and high churn suggest that QPP needs ongoing refinement as value-based payment systems evolve. A limitation of this study is the inability to identify A-APM participants with publicly available data.

## Notes

Supplement 1. **eMethods**.
eReferences

Supplement 2.
Data Sharing Statement
