# Supplementary material for: Seven Years of Participation Churn in the Medicare Quality Payment Program
Source: JAMA Netw Open. 2025 Sep 19;8(9):e2532838. doi: 10.1001/jamanetworkopen.2025.32838 (PMC12449714; doi:10.1001/jamanetworkopen.2025.32838)
Supplement: Supplement 1. — eMethods. eReferences [file jamanetwopen-e2532838-s001.pdf]

Supplemental Online Content

Lin M, Carey K, Gidwani R, Hanchate AD. Seven years of participation churn in Medicare quality payment program. *JAMA Netw Open.* 2025;8(9):e2532838.
doi:10.1001/jamanetworkopen.2025.32838

eMethods.

eReferences

This supplemental material has been provided by the authors to give readers additional information about their work.

© 2025 Lin M et al. *JAMA Network Open*.

eMethods

QPP and Its Two Tracks

The Centers for Medicare & Medicaid Services (CMS) established the Quality Payment Program (QPP) under the Medicare Access and CHIP Reauthorization Act of 2015, shifting Medicare from fee-for-service to value-based payments.[1] Since 2017, Medicare Part B clinicians must participate in QPP, with exceptions for those newly enrolled in Medicare or below the low-volume threshold.[2] Recognizing varying levels of readiness for financial risk among clinicians, CMS created two tracks—Merit-based Incentive Payment System (MIPS) and Advanced Alternative Payment Models (A-APM)—to enable a gradual transition to value-based care.[3]

MIPS serves as a transitional track that links fee-for-service payments to performance metrics based on clinician-reported annual data. Clinicians can report individually, as part of a group practice, virtual group, or through an APM entity ("MIPS APM"). Under MIPS, clinicians receive performance-based payment adjustments two years later without taking on additional financial risk.[4] In contrast, A-APM requires clinicians to accept two-sided risk—higher potential financial rewards along with potential losses.[5] A-APMs are a subset of APMs that carry downside financial risk (requiring repayment to CMS when spending exceeds targets) and use quality metrics similar to those in MIPS. Clinicians in A-APMs who meet minimum volume thresholds (at least 50% of Medicare Part B payments or 35% of Medicare patients through the APM) are exempt from MIPS. Those who don't meet these thresholds or who participate in other APMs fall under the MIPS track (MIPS APM).

The two QPP tracks offer clinicians distinct experiences. MIPS, the default option, serves as a stepping stone for those not yet ready for A-APM. It provides performance-based incentives and focuses on specific measures while requiring detailed reporting but limiting financial risk. A-APM, though requiring two-sided risk, offers greater rewards—including a 5% Part B payment bonus independent of quality performance[6]—and reduces administrative burden. Clinicians can annually choose to maintain their current track, switch between tracks, or modify their reporting mode within MIPS. This flexible framework can lead to fluid participation patterns. Moreover, differences in reporting mechanisms (individual vs. group vs. APM entity) may reflect varying levels of infrastructure, resources, and administrative capacity, potentially influencing clinicians' ability to sustain MIPS participation or transition to A-APMs.

Identification of MIPS Participation

Since public data does not allow identification of A-APM participants, our analysis focuses on the observable aspects of participation churn—specifically, movement in and out of MIPS and changes in reporting modes within MIPS. We categorized each clinician's MIPS participation status as: non-participant, MIPS individual, MIPS group, or MIPS APM. Using the QPP Experience Report,[7] we identified clinicians who participated in the MIPS track and their corresponding reporting mode. Clinicians not listed in the Experience Report were classified as non-MIPS participants, who were either ineligible for QPP or enrolled in an A-APM.

© 2025 Lin M et al. *JAMA Network Open*.

eReferences

1. Doherty RB. Goodbye, Sustainable Growth Rate-Hello, Merit-Based Incentive Payment System. *Ann Intern Med*. 2015;163(2):138-139. doi:10.7326/m15-0992

2. *MIPS Participation Fact Sheet*. Centers for Medicare & Medicaid Services Accessed April 12, 2025. https://www.cms.gov/files/document/qppmipsparticipationfactsheetpdf

3. Quality Payment Program Overview - QPP. Accessed March 7, 2025. https://qpp.cms.gov/about/qpp-overview

4. Traditional MIPS Overview - QPP. Accessed April 12, 2025. https://qpp.cms.gov/mips/traditional-mips

5. Advanced Alternative Payment Models (APMs) - QPP. Accessed April 12, 2025. https://qpp.cms.gov/apms/advanced-apms

6. 2021 Alternative Payment Model (APM) Incentive Payment Fact Sheet. Accessed April 12, 2025. https://www.ama-assn.org/system/files/apm-fact-sheet-hhs-cms.pdf

7. Quality Payment Program Experience. Centers for Medicare & Medicaid Services Data. Accessed March 16, 2024. https://data.cms.gov/jsonapi/node/dataset/b3438273-b4a6-44ca-8fb2-9e6026b74642

© 2025 Lin M et al. *JAMA Network Open*.
