# Supplementary material for: Seven Years of Participation Churn in the Medicare Quality Payment Program
Source: JAMA Netw Open. 2025 Sep 19;8(9):e2532838. doi: 10.1001/jamanetworkopen.2025.32838 (PMC12449714; doi:10.1001/jamanetworkopen.2025.32838)
Supplement: Supplement 2. — Data Sharing Statement [file jamanetwopen-e2532838-s002.pdf]

Data Sharing Statement

Lin. Seven Years of Participation Churn in the Medicare Quality Payment Program. *JAMA Netw Open*. Published September 19, 2025. doi:10.1001/jamanetworkopen.2025.32838

Data
Data available: No

Additional Information
Explanation for why data not available: Data used in the study is publically available.
